# Assessing the Influence of the COVID-19 Pandemic on Gastric Cancer Mortality Risk

**DOI:** 10.3390/jcm13030715

**Published:** 2024-01-26

**Authors:** Yuya Shigenobu, Daisuke Miyamori, Kotaro Ikeda, Shuhei Yoshida, Yuka Kikuchi, Keishi Kanno, Saori Kashima, Masanori Ito

**Affiliations:** 1 Department of General Internal Medicine, Hiroshima University Hospital, 1-2-3 Kasumi, Minamiku, Hiroshima 734-8551, Japan; yshige@hiroshima-u.ac.jp (Y.S.); koikeda@hiroshima-u.ac.jp (K.I.); yoshida.shuhei.0810@gmail.com (S.Y.); cumdeoraboramus1@yahoo.co.jp (Y.K.); kkanno@hiroshima-u.ac.jp (K.K.); maito@hiroshima-u.ac.jp (M.I.); 2 Environmental Health Sciences Laboratory, Graduate School of Advanced Science and Engineering, Hiroshima University, 1-5-1 Kagamiyama, Higashi-Hiroshima 739-8511, Japan; kashima@hiroshima-u.ac.jp; 3 Center for the Planetary Health and Innovation Science, The IDEC Institute, Hiroshima University, 1-5-1 Kagamiyama, Higashi-Hiroshima 739-8511, Japan

**Keywords:** gastric cancer, COVID-19 pandemic, mortality

## Abstract

Background: The global impact of the coronavirus disease 2019 (COVID-19) pandemic on public health has been significant. Upper gastrointestinal endoscopy for screening and diagnosis decreased along with new gastric cancer (GC) diagnoses. Methods: This study assesses how the pandemic affected GC mortality using data from Hiroshima Prefecture, comparing mortality rates between patients diagnosed during the pandemic (2020 and 2021) and pre-pandemic (2018 and 2019) periods. The crude hazard ratios (HRs) and HRs adjusted for age, sex, clinical stage, treatment status, and travel distance to the nearest GC screening facility were estimated using Cox regression models. Subgroup and sensitivity analyses were also performed. Results: A total of 9571 patients were diagnosed, with 4877 eligible for follow-up. The median age was 74 years, and 69% were male. The median follow-up period was 157 days, with events per 1000 person-years at 278 and 374 in the pre-pandemic and pandemic periods, respectively (crude HR, 1.37; adjusted HR, 1.17). The sensitivity and subgroup analyses yielded consistent results. Conclusions: The COVID-19 pandemic increased mortality risk in patients with GC. Further studies are required to observe long-term outcomes and identify the disparities contributing to the increased mortality risk.

## 1. Introduction

The first known case of coronavirus disease 2019 (COVID-19) surfaced in Wuhan, China, in December 2019 [1] and rapidly spread across the globe. In Japan, the initial case was diagnosed in January 2020, prompting the Prime Minister to declare the first state of emergency in April [2]. Subsequently, significant shifts in the behavior of people emerged as they refrained from seeking medical examinations, resulting in a profound impact on public health [3,4].

The COVID-19 pandemic led to delays in cancer diagnosis and treatment, often resulting in more advanced stages of cancer [5,6,7,8]. Gastric cancer (GC) stands as a substantial global health concern and is the fifth leading type of cancer and the fourth leading cause of cancer deaths worldwide, accounting for 768,793 deaths in 2020 [9]. Despite declining mortality rates, GC remains the second and fourth leading cause of cancer-related death in Japanese men and women, respectively, with 26,455 and 14,256 deaths recorded in 2022 [10]. Upper gastrointestinal endoscopy plays a pivotal role in GC diagnosis as it combines both gross observations and pathological evaluation via biopsy. The 2014 edition of the Japanese Guidelines for Gastric Cancer Screening recommends biennial endoscopic screening for individuals aged >50 years [11].

In the early stages of the pandemic, the Ministry of Health, Labor and Welfare recommended suspending cancer screening to prioritize COVID-19 treatment and prevent infections in Japan [12]. Furthermore, there were concerns about medical staff facing an increased risk of exposure to aerosol contamination during gastrointestinal endoscopies [13]. Consequently, the Japan Gastroenterological Endoscopy Society recommended the postponement of nonurgent gastrointestinal endoscopies [14]. Coupled with the reluctance of the general population to visit healthcare professionals unless seriously ill due to fear of infection [15], the number of upper gastrointestinal endoscopies for both screening and diagnosis decreased by up to 42.1% [16]. This decline corresponded with a significant drop in newly diagnosed GC cases, up to 73.2% [7]. Notably, we found a decline in the number of patients diagnosed with stage I disease and an increase in patients diagnosed with stage IV disease [17]. Furthermore, a previous study indicated a reduction in GC surgeries during the COVID-19 pandemic in Japan, particularly distal gastrectomy, which was 81% of the pre-pandemic level [18,19]. Similar trends in GC patients have been documented in other countries. A study involving 145 centers across 50 countries revealed a progression in clinical staging, an uptick in cases with distant metastases, and a reduction in the number of surgical interventions [20].

Some studies have explored the possibility of increased GC-related mortality risk during the COVID-19 pandemic; however, the results remain controversial. A retrospective study conducted at a single center in Portugal reported an uptick in mortality among GC patients following the pandemic [21]. In contrast, a retrospective study at a single center in Israel found no significant change in GC-related mortality [22]. In a cohort study conducted in Ontario, Canada, investigating the short-term survival rates of newly diagnosed cancer patients during the COVID-19 pandemic, no significant association was observed between GC mortality and the pandemic. Notably, the study encompassed various cancer types and did not specifically focus on GC. Furthermore, the impact of the clinical stage at diagnosis, a crucial factor in cancer outcomes, was not taken into consideration [23]. Moreover, all of these studies included patients diagnosed with GC in 2020 and not those diagnosed in 2021; due to the ongoing COVID-19 pandemic in 2021, there is a possibility that the short-term prognosis for these patients may have been influenced. Additionally, traveling long distances to screening facilities reportedly increased diagnostic delay and cancer mortality risk [23,24,25]. The pandemic, with its mobility restrictions and decreased travel [26], could have influenced outcomes, potentially varying based on proximity to screening facilities.

We aimed to assess the impact of the COVID-19 pandemic on GC mortality rates and to identify contributing factors, utilizing data from a large cancer registry database in Hiroshima Prefecture.

## 2. Materials and Methods

### 2.1. Study Design

This retrospective cohort study was conducted in Hiroshima Prefecture. Hiroshima Prefecture has the 12th largest population among Japan’s 47 prefectures, which was 2.78 million in 2022. In Japan, each prefecture designates specific hospitals as cancer centers to ensure specialized medical care and collaboration in cancer care. In Hiroshima Prefecture, 15 hospitals, including one university hospital, one cancer hospital, and 13 cancer-designated hospitals, hold this designation. The Hiroshima Cancer Medicine Collaboration Council Institutional Cancer Registration Subcommittee Cancer Registry database collects data on patients with cancer in these hospitals. This database contains information on patients newly diagnosed with cancer, including demographics, histopathology, clinical stage, treatment status, mortality, and time from diagnosis to death. For this study, we analyzed GC data between 2018 and 2021 within this database. The study protocol was approved by the Ethical Committee for Clinical Research of Hiroshima University (E2022-0139) on 2 November 2022, and was performed in accordance with the principles of the Declaration of Helsinki.

### 2.2. Inclusion Criteria

Patients who received a new diagnosis of GC between 2018 and 2021 and were registered in the database were included. GC was defined in accordance with the International Classification of Diseases for Oncology, Third Edition (ICD-O-3), with a site code of C16 (Supplementary Table S1) and histology codes detailed in Supplementary Table S2, including adenocarcinoma, carcinoid tumor, gastrointestinal stromal tumor, squamous cell carcinoma, and neoplasm [27].

### 2.3. Exposures

In this study, we defined the pandemic period as the exposure period and the pre-pandemic period as the control period. Japan declared its first state of emergency on 7 April 2020, and on 26 April 2020, the Ministry of Health, Labor, and Welfare requested a delay in cancer screening [12]. Although this request was retracted on 26 May 2020, the Japan Gastroenterological Endoscopy Society recommended the postponement of non-urgent gastrointestinal endoscopies [14]. Consequently, the number of upper gastrointestinal endoscopies performed to diagnose GC decreased by 9.4% to 38.6% between April 2020 and January 2021 [16]. Considering these factors, we defined the pandemic period as 2020–2021 and the pre-pandemic period as 2018–2019. Patients were assigned to either period according to the date of diagnosis.

### 2.4. Outcomes

The primary outcome was mortality from any cause among patients diagnosed with GC between 2018 and 2021 because the database did not provide a clear reason for fatalities.

### 2.5. Covariates

Covariates included sex, age, clinical stage at diagnosis, treatment status, histological findings, and travel distance between the patient’s residence and the nearest GC screening facility. The clinical stage was classified according to the 8th edition of the Union for International Cancer Control. We used clinical stages because confirmation of pathological stages requires surgical treatment. Treatment status was evaluated with and without treatment, such as open surgery, laparoscopy, endoscopy, radiotherapy, and chemotherapy. Tumor histological findings were classified according to the ICD-O-3.

#### Travel Distance to the GC Screening Facilities

Travel distance is often considered a more precise measure of accessibility [24,28]. To determine the travel distance from the patient’s residence to the nearest GC screening facilities, we employed Esri ArcGIS Pro 3.1 for the following processes. As the database only provided zip codes for the patients’ residences, the centroid of each zip code served as a proxy. These zip codes were then converted to latitude and longitude and plotted on a GIS map. The locations of GC screening facilities and cancer hospitals were obtained from the medical checkup information pages of the official websites of Hiroshima Prefecture [29] and plotted similarly after converting their addresses. We then calculated the travel distances by car using network analysis on the road network [30]. The travel distance for GC patients who lived on islands and needed to travel by passenger boat to the nearest screening facility was calculated using GIS maps provided by the Ministry of Land, Infrastructure, Transport and Tourism. The calculated travel distances were divided into quartiles and incorporated into the following analysis.

### 2.6. Statistical Analysis

All statistical analyses were performed using Stata version 17 MP software (StataCorp LLC, College Station, TX, USA). First, we described the characteristics of GC patients diagnosed during the pre-pandemic and pandemic periods, respectively. Survival analysis was then performed. In our study, the survival period was determined based on the date of GC diagnosis. To highlight its novelty, the follow-up period was set to 1 year after GC diagnosis. We defined censoring as patients who were lost to follow-up owing to missing medical records or were still alive at the end of the follow-up period. In our analysis, all-cause mortality was a censoring event. Kaplan–Meier curves were generated to compare overall mortality between the control and exposure periods using a log-rank test. Schoenfeld residuals were employed to assess the propositional hazard assumptions. For the main analysis, Cox proportional hazards regression was utilized and two models were employed: (1) a crude model and (2) a multivariable-adjusted model, which included age, sex, clinical stage at diagnosis, treatment status, and travel distance. Additionally, two sensitivity analyses were performed: (1) comparing each year to 2018, serving as the reference period, and (2) defining the pre-pandemic period as January 2018–June 2019 and the pandemic period as July 2019–December 2021, because GC patients newly diagnosed in the second half of 2019 were considered more likely to receive treatment during the COVID-19 pandemic. Subgroup analysis was carried out for each covariate. Similar to the main analysis, crude and multivariable-adjusted models, which included covariates other than the subgroup itself, were employed. Additionally, a likelihood ratio test was performed to assess the goodness of fit, ensuring robustness in each subgroup.

## 3. Results

### 3.1. The Flow Chart

**Figure 1:**
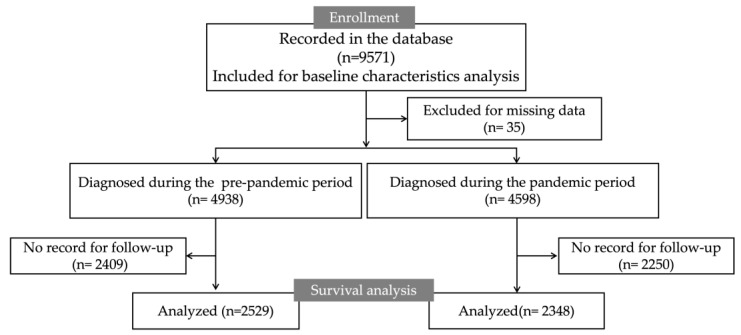
The flow chart.

Figure 1 illustrates a flowchart depicting the cohort inclusion/exclusion criteria. Initially, a total of 9571 patients recorded in the database were enrolled, and 35 were excluded owing to missing data. In the pandemic and the pre-pandemic periods, 4598 and 4938 patients were diagnosed with GC, respectively. During the pre-pandemic period, follow-up records were missing for 2409 cases, leaving 2529 cases for survival analysis. During the pandemic period, follow-up records were missing for 2250 patients, resulting in 2348 patients for survival analysis.

### 3.2. Summary of Baseline Characteristics

Table 1 summarizes the baseline characteristics of the patients. The mean age (interquartile range) of patients in the pre-pandemic and pandemic periods was 75 (68–81) and 74 (68–81) years, respectively. The percentage of males was 69% in the pre-pandemic period and 70% in the pandemic period, respectively. Clinical stages at diagnosis, from stage I to stage IV, were 65%, 8%, 7%, and 16% in the pre-pandemic period and 64%, 7%, 6%, and 17% in the pandemic period, respectively. Regarding treatment status, 45% of the patients received endoscopic treatment, 21% underwent laparoscopic surgery, and 14% had open surgery in the pre-pandemic period, and in the pandemic period, the percentages were 44%, 22%, and 15%, respectively. The percentage of patients who did not receive treatment was 15% during the pre-pandemic period and 14% during the pandemic period. In both the pandemic and pre-pandemic periods, more than 90% of patients were diagnosed with adenocarcinoma. The travel distance to the nearest GC screening facilities was 12 m for the closest patient and 30,475 m for the farthest. The percentage of patients who lived within a travel distance of 609 m from the screening facility was 25% in both periods. The percentage of patients who live within a travel distance of 610–1063 m changed from 26% to 24%, whereas the percentage of patients who lived farther away from the facility, with a travel distance of 2225 m or more, changed from 23% to 26%.

### 3.3. Survival Analysis and Hazard Ratios (HRs) in Patients with GC during the COVID-19 Pandemic

**Figure 2:**
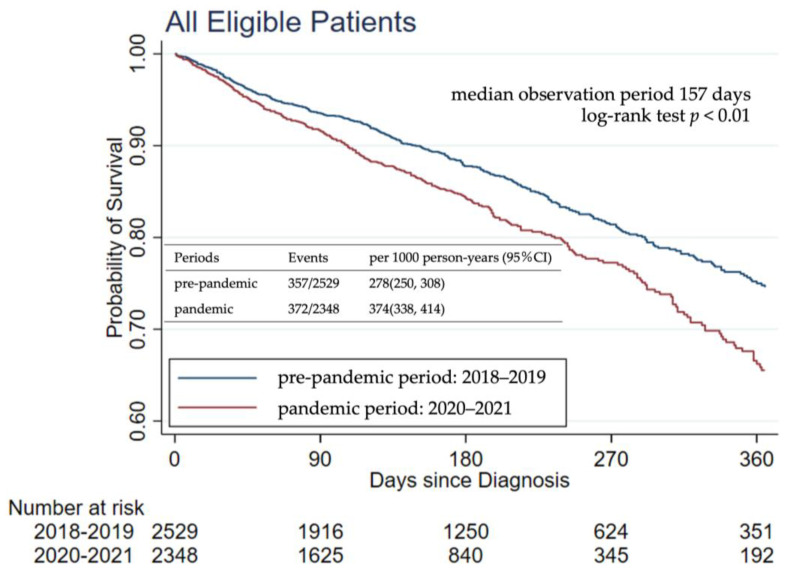
Kaplan–Meier curves for overall survival in the main analysis.

Figure 2 displays the Kaplan–Meier curves for estimating the overall survival of patients with GC. The median observation period was 157 days. The number of events was 357 out of 2529 (278 events per 1000 person-years, 95% confidence interval [CI]: 250–308 events per 1000 person-years) in the pre-pandemic period and 372 out of 2348 (374 events per 1000 person-years, 95% CI: 338–414 events per 1000 person-years) in the pandemic period. The Schoenfeld residuals test indicates no violation of the proportional hazards assumption between exposure and control periods (*p* = 0.51). The results of the Cox proportional hazards regression model for the main analysis and the two sensitivity analyses are presented in Table 2. In the pandemic period, when compared with the pre-pandemic period, the crude HR and HR adjusted for age, sex, clinical stage, treatment status, and travel distance were 1.37 (95% CI: 1.18–1.58) and 1.17 (95% CI: 1.01–1.36), respectively. HRs for the pandemic period approached null after adjusting for covariates.

Two sensitivity analyses were performed to examine the impact of different cutoff values in defining the pre-pandemic and pandemic periods. The first sensitivity analysis compared the survival rates of patients diagnosed in 2019, 2020, and 2021 with those in the reference year, 2018. Kaplan–Meier curves were used to estimate the overall survival of patients with GC (Supplementary Figure S1). Patients diagnosed in 2020 and 2021 had a significantly higher mortality risk than those diagnosed in 2018 (2020, crude HR: 1.47, 95% CI: 1.19–1.81, 2021, crude HR: 1.40, 95% CI: 1.14–1.73). The second sensitivity analysis defined the pre-pandemic period from January 2018 to June 2019 and the pandemic period from July 2019 to December 2021. It revealed that the mortality risk of patients diagnosed in the pandemic period was significantly higher than that of patients diagnosed in the pre-pandemic period (crude HR: 1.36, 95% CI: 1.17–1.58, adjusted HR: 1.26, 95% CI: 1.08–1.47). Importantly, no violations of the proportional hazards assumption were observed in the main or sensitivity analyses.

Kaplan–Meier curves show the estimate of the probability of survival from all-cause deaths among patients with GC. The Schoenfeld residuals test indicates no violation of the proportional hazards assumption between exposure and control periods (*p* = 0.51).

### 3.4. Subgroup Analysis of the Impact of the Pandemic Period on GC Mortality Risk

**Figure 3:**
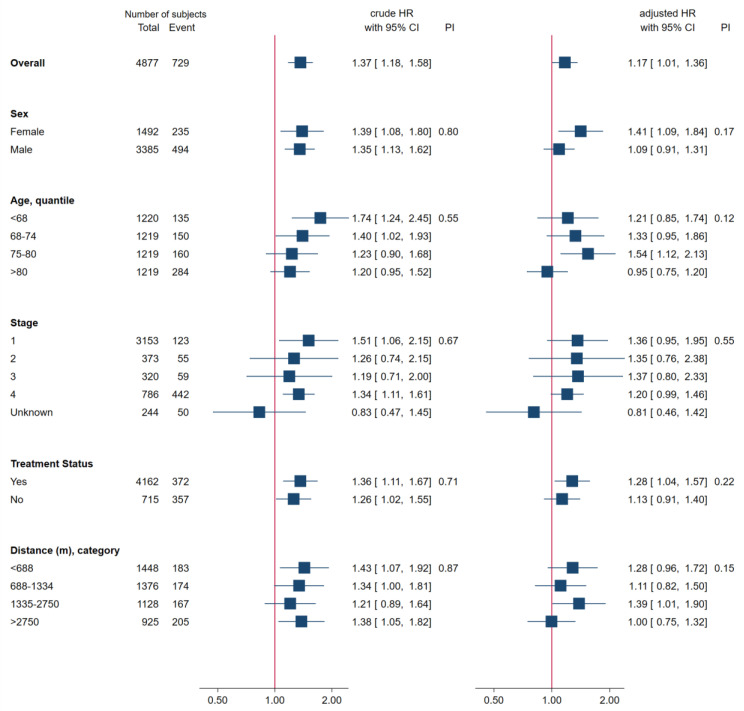
Subgroup Analysis.

A subgroup analysis was conducted to explore the impact of the pandemic period on GC mortality risk (Figure 3). Subgroups were based on sex, age, clinical stage, treatment status, and travel distance. Age and travel distance were divided into quartiles. The vertical line represents a hazard ratio of one. The forest plots exhibit two different HR values: one crude on the left and the other adjusted on the right. The results consistently indicated an increase in mortality risk across all subgroups during the pandemic period. Furthermore, a likelihood ratio test revealed no significant interactions between the pandemic period and any subgroup category in the crude and adjusted models, suggesting that demographic and clinical factors did not substantially affect the impact of the pandemic period on GC mortality risk.

## 4. Discussion

In this study, we examined the impact of the COVID-19 pandemic on the risk of GC mortality using a comprehensive cancer registry database from Hiroshima Prefecture. During the COVID-19 pandemic, the mortality risk was 1.4 times higher than that during the pre-pandemic period. This result remained statistically significant even after adjusting for clinical stage at diagnosis, treatment status, histological findings, travel distance, and sensitivity analyses. Although the adjusted HR was significant, it approached null, indicating that covariates played a role in the increased risk of GC mortality due to the COVID-19 pandemic.

Notably, treatment outcomes for GC have been improving in Japan, with the 5-year survival rate increasing from 61.6% (1993–1996) to 66.6% (2009–2011) [31,32]. However, our study revealed that patients diagnosed with GC during the COVID-19 pandemic experienced lower survival rates, highlighting the significant impact of the pandemic on GC prognosis. While some studies have evaluated the impact of the COVID-19 pandemic on mortality risk, specifically for GC, the results remain controversial. A single-center retrospective study of a cancer hospital in Portugal showed increased mortality in patients with GC after the pandemic [21], whereas a single-center retrospective study of general hospitals in Israel showed no change in the risk of death from GC [22]. However, the statistical power of these studies was limited owing to small sample sizes. A study utilizing an extensive database from northeastern Spain revealed a decrease in the incidence of GC; however, the mortality remained unchanged from 2019 to 2020 [33]. Notably, in this previous study, patients who succumbed to the disease were not necessarily diagnosed during the COVID-19 pandemic. This distinction sets it apart from our investigation, which specifically examined one-year mortality rates for patients diagnosed with GC during the pandemic. Our study utilized a community-based database encompassing all cancer hospitals in Hiroshima Prefecture, Japan, and analyzed patients diagnosed with GC from 2018 to 2021. This enabled us to present a more precise depiction of the current scenario and demonstrate a substantial increase in the risk of GC mortality due to the COVID-19 pandemic.

A previous multicenter retrospective cohort study in Japan reported a 32.9% decrease in stage I cases and an 11.4% increase in stage IV cases of GC during the COVID-19 pandemic [7]. If left untreated, early-stage GC progresses to advanced cancer within 34–44 months [34]. Furthermore, the 5-year survival rate for stage I is >90%, but drops to 45% for stage III, and is only 9% for stage IV [35]. In this study, the percentage of stage IV changed from 16% to 17% during the pandemic period, and unknown cases also changed from 4% to 6%. Furthermore, the percentage of patients who underwent laparoscopic surgery changed from 22% to 20%, and those who received open surgery changed from 15% to 13%. These changes are consistent with reports that the pandemic has reduced the number of cases that can be surgically cured [36,37]. This study revealed a decrease in early-stage cases and an increase in advanced and unknown cases, aligning with reports that fewer patients underwent thorough examinations or biopsies to confirm a diagnosis [38].

We hypothesized that behavioral changes due to the COVID-19 pandemic could affect GC mortality rates, particularly in areas with limited medical resources and greater distances to screening facilities. However, our study found no relation between the pandemic and travel distance in terms of GC mortality. This may indicate that COVID-19 is less prevalent in rural areas and more likely to induce behavioral changes in urban areas [39,40,41]. In our study, the percentage of patients with short travel distances decreased from 26% to 24%, potentially increasing mortality risk.

In this study, the adjusted HR approached null compared with the crude HR but was still significant. The database did not include important prognostic factors such as comorbidities, physical activity, performance status, and smoking and could not be incorporated into the adjusted model. The adjustment model included chemotherapy and radiation therapy but did not account for interruptions or changes. In addition, previous reports indicate prolonged waiting times from diagnosis to surgery and delays in the initiation of chemotherapy due to the pandemic, and our study did not include an evaluation of these aspects [42]. However, various studies have shown that the wait time between diagnosis and treatment does not affect the prognosis of GC [43,44,45,46]. Although there are concerns about changes in the quality of treatment due to the COVID-19 pandemic, reports in Japan have shown no changes in the postoperative 30- or 90-day mortality rates or in the incidence of postoperative complications such as pneumonia and sepsis [19,47]. Additionally, we could not investigate the impact of psychological stress associated with the pandemic on the risk of GC mortality. Conducting further studies that include these factors to identify possible intervention risks is crucial. According to the Comprehensive Survey of Living Conditions, the uptake rate for GC screening in Japan had reverted to pre-pandemic levels by 2022 [48]. Therefore, conducting a comparison and analysis with short-term prognoses of GC patients diagnosed after 2022 may aid in identifying the factors that contributed to the increased mortality during the pandemic period.

This study has several strengths. Using a large database covering all newly diagnosed patients with GC in Hiroshima Prefecture allowed us to evaluate the most critical outcome, death, in the at-risk population. Despite the short 1-year follow-up period, mortality risk from GC significantly increased, indicating the substantial impact of the pandemic. While previous studies primarily focused on patients diagnosed until the year 2020, our research extends its scope to include GC patients diagnosed in 2021. Sensitivity analysis, utilizing patients diagnosed in 2018 as a reference, revealed a statistically significant increase in mortality rates among those diagnosed in 2021. This finding substantiates more robustly the notion that the COVID-19 pandemic has adversely impacted the short-term prognosis of GC. We also found that cancer progression, changes in treatment, and changes in travel distance to the nearest GC screening facilities were responsible for the increased risk of GC during the pandemic.

Nevertheless, this study has some limitations. Firstly, the possibility of selection bias among the periods that were followed up compared with those that were not is crucial to consider. Follow-up rates varied among hospitals because cancer hospitals were required to register all patients diagnosed with GC, yet the follow-up period for each patient was extended to each hospital. Although follow-up rates varied by hospital, this variation remained constant between the periods. Secondly, the database lacked information regarding the specific cause of death and comorbidities, making it possible that some of the outcomes were attributed to COVID-19-related mortality. Notably, patients undergoing chemotherapy for cancer are known to face an elevated risk of succumbing to COVID-19 [21]. However, the cumulative incidence of COVID-19 in Hiroshima Prefecture up to the conclusion of 2021 stood at 22,221 new cases and 202 deaths, representing only 0.8% and 0.007% of the population, respectively (Supplementary Figure S2). Consequently, the direct impact of COVID-19 on the mortality of patients with GC is likely to be limited. Lastly, because this study was conducted within the framework of the Japanese healthcare system, all of the findings may not be directly applicable to healthcare systems with different resource availability. However, a number of countries have imposed stricter policies than those of Japan, including lockdowns, behavioral restrictions, and zero-COVID policies. Therefore, in those countries, the impact of the pandemic on GC mortality may be relatively more significant than in Japan. This study can inform healthcare policymakers about potential challenges and guide them in developing targeted strategies to address the impact on cancer outcomes during a pandemic.

## 5. Conclusions

In conclusion, this study provides compelling evidence linking the COVID-19 pandemic to a heightened risk of GC mortality. Various factors, including age, sex, clinical stage at diagnosis, treatment status, and proximity to the nearest GC screening facility, contributed to this increased risk during the pandemic. While this study sheds light on these factors, it is important to acknowledge that we may not have captured all relevant variables. Further investigations with a focus on long-term outcomes are imperative to gain a more comprehensive understanding of the impact of the COVID-19 pandemic on GC outcomes. These insights will be crucial for shaping targeted interventions and healthcare policies to mitigate the consequences of future public health crises on cancer care.

## Figures and Tables

**Table 1:** Baseline characteristics.

		Total		Pre-Pandemic Period		Pandemic Period	
		N = 4877		N = 2529		N = 2348	
Age, Median (IQR)-yr		74	(68–81)	74	(68–80)	75	(68–81)
Male sex, No (%)		3385	(69)	1750	(69)	1635	(70)
Clinical stage, No (%)							
	0	1	(0)	1	(0)	0 (0)	(0)
	I	3153	(65)	1648	(65)	1505	(64)
	II	373	(8)	201	(8)	172	(7)
	III	320	(7)	176	(7)	144	(6)
	IV	786	(16)	392	(16)	394	(17)
	Unknown	244	(5)	111	(4)	133	(6)
Histology, No (%)							
	Adenocarcinoma	4586	(94)	2380	(94)	2206	(94)
	Carcinoids	73	(1)	34	(1)	39	(2)
	GIST	142	(3)	76	(3)	66	(3)
	Squamous cell carcinoma	5	(0)	2	(0)	3	(0)
	Neoplasms, NOS	71	(1)	37	(1)	34	(1)
Treatment status, No (%)							
	Endoscopic treatment	2180	(45)	1108	(44)	1072	(46)
	Laparoscopic surgery	1031	(21)	558	(22)	473	(20)
	Open surgery	689	(14)	382	(15)	307	(13)
	Radiotherapy	45	(1)	11	(0)	34	(1)
	Chemotherapy	890	(18)	465	(18)	425	(18)
	Endocrine therapy	3	(0)	1	(0)	2	(0)
	Other therapy	3	(0)	1	(0)	2	(0)
	No treatment recorded	715	(15)	361	(14)	354	(15)
Travel distance, No (%)							
	<609 m	1224	(25)	633	(25)	591	(25)
	610–1063 m	1221	(25)	656	(26)	565	(24)
	1065–2225 m	1222	(25)	646	(26)	576	(25)
	2225 m<	1210	(25)	594	(23)	616	(26)

IQR: interquartile range, NOS: not otherwise specified.

**Table 2:** Crude and adjusted HR from all-cause mortality among patients with GC according to different cutoffs between pandemic and pre-pandemic periods.

	Periods	HR	95% CI	Adjusted HR *	95% CI
Main analysis	pre-pandemic	Ref		Ref	
	pandemic	1.37	1.18, 1.58	1.17	1.01, 1.36
Sensitivity analysis 1	2018	Ref		Ref	
	2019	1.10	0.89, 1.36	1.08	0.88, 1.3
	2020	1.47	1.19, 1.81	1.20	0.97, 1.49
	2021	1.40	1.14, 1.73	1.23	1.00, 1.52
Sensitivity analysis 2	Jan. 2018 to Jun. 2019	Ref		Ref	
	Jul. 2019 to Dec. 2021	1.36	1.17, 1.58	1.26	1.08, 1.47

* Adjusted for age, sex, clinical stage at diagnosis, treatment status, and travel distance. Test for proportional hazard assumptions was not violated for the main and sensitivity analysis via the Schoenfeld residual test (*p* = 0.62 and *p* = 0.25, respectively). HR: hazard ratio; CI: confidence interval; Ref: reference; Jan: January; Jun: June; Jul: July; Dec: December.

## Data Availability

Data can be used upon reasonable request.

## Supplementary Materials

The following supporting information can be downloaded at https://www.mdpi.com/article/10.3390/jcm13030715/s1, Figure S1: Overall survival analysis for year-to-year comparisons using 2018 as the reference year; Figure S2: The cumulative number of COVID-19 cases and deaths; Table S1: ICD-O-3 site codes included in this study; Table S2: ICD-O-3 histology codes included in this study.
