# Supplementary material for: Assessing the Influence of the COVID-19 Pandemic on Gastric Cancer Mortality Risk
Source: J Clin Med. 2024 Jan 26;13(3):715. doi: 10.3390/jcm13030715 (PMC10856106; doi:10.3390/jcm13030715)
Supplement: Supplementary file 1 [file jcm-13-00715-s001.zip › jcm-2771766-supplementary.pdf]

Supplementary Materials

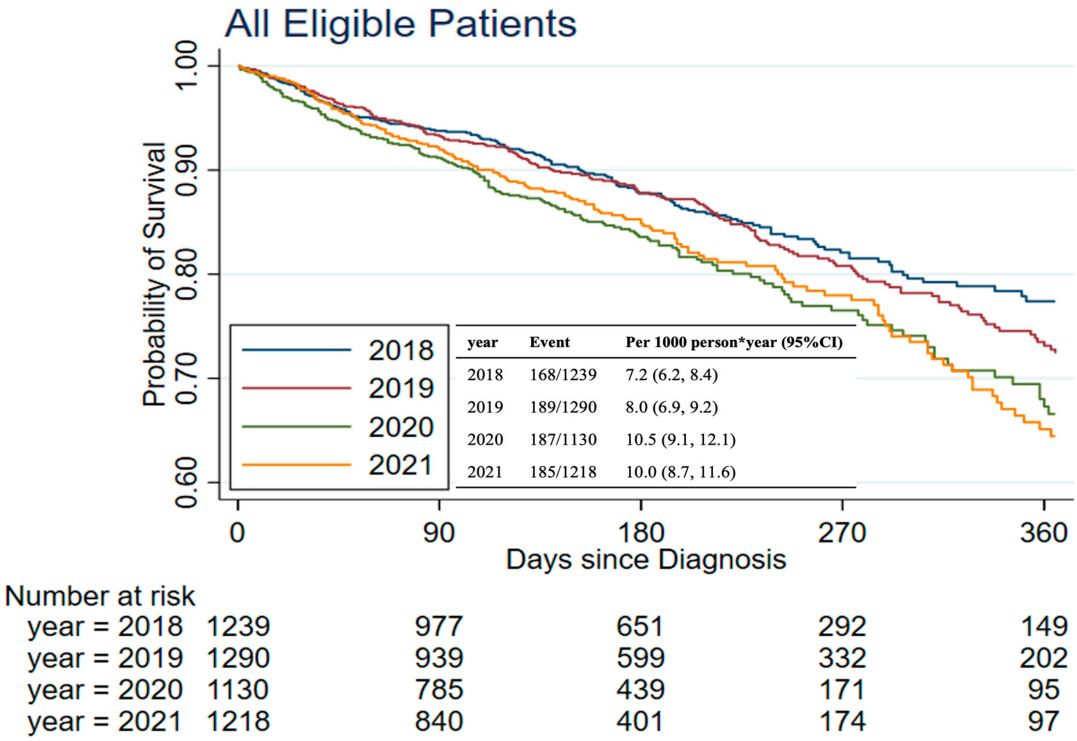

Figure S1. Overall survival analysis for year-to-year comparisons using 2018 as the reference year.

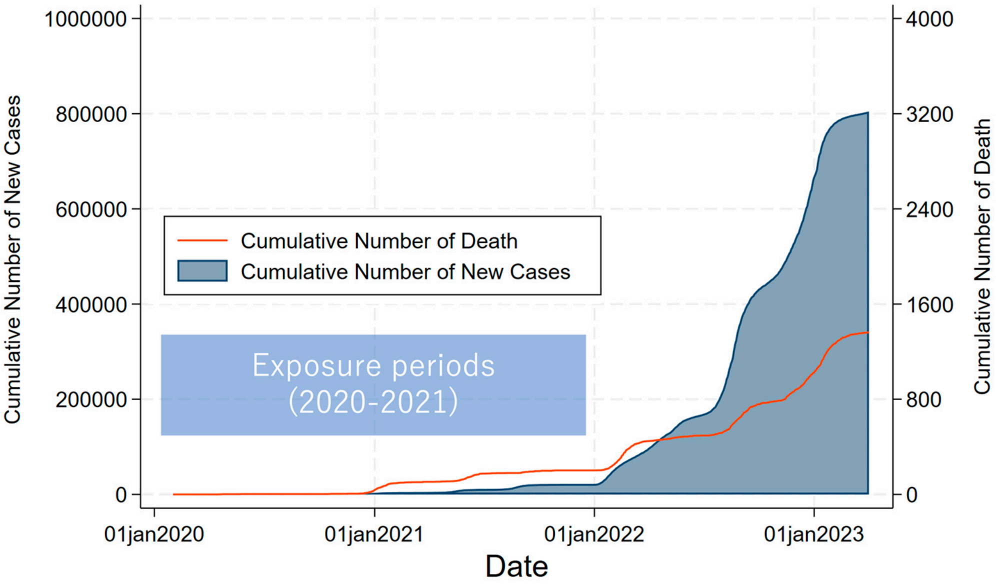

Figure S2. The Cumulative Number of COVID-19 Cases and Deaths. The figure illustrates the cumulative number of COVID-19 cases and deaths. As of 2021, the population of Hiroshima Prefecture is 2.81 million. There have been 22,221 new cases and 202 deaths due to COVID-19, which represent 0.8% and 0.007% of the population, respectively.

Table S1: ICD-O-3 Site codes included in this study

ICD-O-3 Site Codes	Site
C16.0	Cardia, NOS
C16.1	Fundus of stomach
C16.2	Body of stomach
C16.3	Gastric antrum
C16.4	Pylorus
C16.5	Lesser curvature of stomach, NOS (no classifiable to C16.1 to C16.4)
C16.6	Greater curvature of stomach, NOS (no classifiable to C16.0 to C16.4)
C16.8	Overlapping lesion of stomach
C16.9	Stomach, NOS

NOS: not otherwise specified.

Table S2: ICD-O-3 histology codes included in this study

Subtypes	ICD-O-3 Histology Codes
Adenocarcinoma	8140/8145 8147 8210 8211 8214 8220 8221 8230 8231 8255 8260/8263 8310 8480 8481 8490 8510 8560 8562 8570 8576
Carcinoid	8013 8154 8240/8246 8249
GISTs	8936
Squamous cell carcinoma	8070 8083
Neoplasm, NOS	8000 8010 8020

NOS: not otherwise specified.
